# CUB and Sushi Multiple Domains (CSMD1) Gene Polymorphisms and Susceptibilities to Idiopathic Parkinson's Disease in Northern Chinese Han Population: A Case-Control Study

**DOI:** 10.1155/2021/6661162

**Published:** 2021-02-12

**Authors:** Xinling Bai, Jianing Jin, Shanshan Li, Huimin Wang, Anmu Xie

**Affiliations:** ^1^Department of Neurology, Affiliated Hospital of Qingdao University, Qingdao, China; ^2^Neurological Regulation Institute of Qingdao University, Qingdao, China

## Abstract

Evidence has shown that the CUB and Sushi Multiple Domains (*CSMD1*) gene is an inhibitor of the complement activation pathway and is also involved in central nervous system inflammation. Previous studies have revealed that the *CSMD1* gene is related to familial Parkinson's disease. This study aimed to investigate the relationship between *CSMD1* gene and susceptibility to Parkinson's disease in population of northern China. A case-control study was performed on 423 Parkinson's disease patients and 465 healthy controls matched for age and sex. DNA from enrolled subjects were extracted from the peripheral blood, and single nucleotide polymorphisms (SNPs) rs12681349 (C＞T), rs10503253 (C＞A), and rs1983474 (T＞G) within CSMD1 gene were genotyped using polymerase chain reaction-restriction fragment length polymorphism (PCR-RFLP). Genotype frequency of rs10503253 (CA versus CC : OR = 1.554, 95% CI = 1.169–2.066, *p*=0.002) and rs1983474 (GG versus TT : OR = 0.599, 95% CI = 0.401–0.895, *p*=0.012) was significantly different between PD cases and controls, but not for rs12681349. Comprehensive and subgroup analysis indicated that rs10503252 showed significant statistical differences in the dominant model (AA + CA versus CC : OR = 0.677, 95% CI = 0.517–0.886, *p*=0.004), late-onset cohort (CA versus CC : OR = 1.570, 95% CI = 1.159–2.126, *p*=0.004), and the female cohort (CA versus CC : OR = 0.687, 95% CI = 0.497–0.952, *p*=0.023), compared with the matched control group. The difference of recessive model of rs1983474 (GG versus TT + TG : OR = 1.837, 95% CI = 1.287–2.620, *p*=0.001) was significant in Parkinson's disease. According to the subgroup analysis, results indicated that late-onset cohort (GG versus TT : OR = 0.643, 95% CI = 0.420–0.985, *p*=0.042), male cohort (TG versus TT : OR = 2.160, 95% CI = 1.162–4.016, *p*=0.015), and female group (GG versus TT : OR = 0.418, 95% CI = 0.234–0.746, *p*=0.003) of rs1983474 were significantly associated with Parkinson's disease susceptibility. In both genotype and subgroup analysis, we failed to find any relationship between rs12681349 polymorphism and Parkinson's disease risk. Our results indicate that the rs10503253 and rs1983474 gene polymorphism may be associated with idiopathic Parkinson's disease susceptibility in Chinese population. Nevertheless, these conclusions need to be further verified by more studies.

## 1. Introduction

Parkinson's disease (PD) is a common progressive neurodegenerative disease second only to Alzheimer's disease, affecting about 1-2% of people over the age of 65 years [1, 2]. The main clinical symptoms of PD are quiescent tremor, bradykinesia, ankyloses, and postural instability. Meanwhile, nonmotor symptoms of PD are getting more and more attention, including cognitive impairment, depression, anxiety, and autonomic nerve dysfunction, and olfaction disorder [3, 4]. Interestingly, females are less likely to develop PD and studies have found that estrogen in females can reduce the dysfunction of dopaminergic neurons in PD patients [5]. However, the etiology and pathogenesis of Parkinson's disease are unclear. Beyond the mutations of candidate genes, the interaction between susceptibility genes and environmental factors plays an important role in the complex etiology of PD. Genome-wide association study (*GWAS*) has contributed to the discovery of genes associated with PD and other lesser-known pathways, including inflammation and immune dysfunction, dysregulation, transcription, vascular pathology, and neurotransmitter [6].

*CSMD1* gene (OMIM ID: 608397) spanning at least 1 Mb on chromosome 8p23.2 [7] is considered to be an inhibitor of complement activation and a regulator of inflammation in the developing central nervous system and highly expressed in the central nervous system [8]. The complement cascade not only involves defending against pathogens in the immune system but also plays a crucial role in synaptic pruning and synaptic plasticity [9], which seem to be involved in cognitive functions and psychiatric disorders [10]. A previous *GWAS* [11] suggested that *CSMD1* gene was significantly associated with PD risk. In addition, a complete exon sequencing in Spanish population showed a link between mutations in the *CSMD1* gene and familial Parkinson's disease [12]. Based on the previous studies and the close relationship between *CSMD1* gene with complement pathway and inflammatory response, we hypothesized that *CSMD1* may be a candidate gene for idiopathic Parkinson's disease. The *CSMD1* variant rs10503253, the SNP, has been reported to be associated with cognitive function and executive ability in healthy individuals [13]. Another polymorphism of rs12681349 within *CSMD1* has been explored in Iranian population [14]. The rs1983474 within *CSMD1* has been revealed to be connected with variation in urinary C-telopeptide of type II collagen (u CTX-II) levels in patients with osteoarthritis [15]. The above three SNPs are all missense mutation points of *CSMD1* gene (NCBI: http://www.ncbi.nlm.nih.gov/SNP/snp_ref.cgi?locusId=64478). However, they have never been studied in Chinese Parkinson's disease population.

The relationship between *CSMD1* gene polymorphisms and PD sensitivity has already been investigated in some countries, but not China. In consequence, we conducted a case-control study to explore the risk relevance between three SNPs (rs12681349, rs10503253, and rs1983474) of *CSMD1* gene with PD in Northern Chinese aiming to provide new insights into the pathogenesis of PD and generate ideas for early diagnosis and treatment of PD.

## 2. Materials and Methods

### 2.1. Case Selection

We selected a case-control study on 423 cases and 465 healthy controls. The demographics of the cohorts are given in Table 1. Patients diagnosed with Parkinson's disease by two neurologists according to the revised clinical diagnostic criteria were enrolled from the Neurology Clinic of the Affiliated Hospital of Qingdao University, China, from January 2016 to March 2019. The clinical diagnostic criteria for Parkinson's disease are bradykinesia in combination with either rest tremor, rigidity, or both, no absolute exclusion criteria, at least two support criteria, and no warning signs [16]. None of the patients had a family history of Parkinsonism, neuropathy, or psychosis conditions and none of them were related. Healthy controls were selected from the same hospital physical examination center. The exclusion criteria were Parkinson's disease, Alzheimer's disease, dementia, stroke and epilepsy, diabetes, hypertension, or other Parkinson's disease risk-related diseases. This study was verified and approved by the Ethics Committee of the Affiliated Hospital of Qingdao University. All subjects gave signed informed consent before blood collection and the study was performed according to the national ethical standards and the Code of Ethics of the World Medical Association (Declaration of Helsinki) for human genome research.

### 2.2. Sample Collection

2 ml peripheral venous blood was collected in 0.5% EDTA anticoagulant tube and stored at −20°C. Whole blood genomic DNA was extracted using DNA extraction kit (TIANGEN Biotech (Beijing) Co., Ltd.) and stored at −20°C according to instructions.

### 2.3. SNPs Selection

The three SNPs (rs12681349, rs10503253, and rs1983474) of CSMD1 gene were selected from NCBI database (http://www.ncbi.nlm.nih.gov/projects/SNP) and SNPinfo (https://snpinfo.niehs.nih.gov/snpfunc/.htm). Genome-wide association studies (http://pdgene.org/) have shown that allele frequencies of above of them were ≥0.2. Their functions were captured in the published literature.

### 2.4. Genotyping

**Figure 1:**
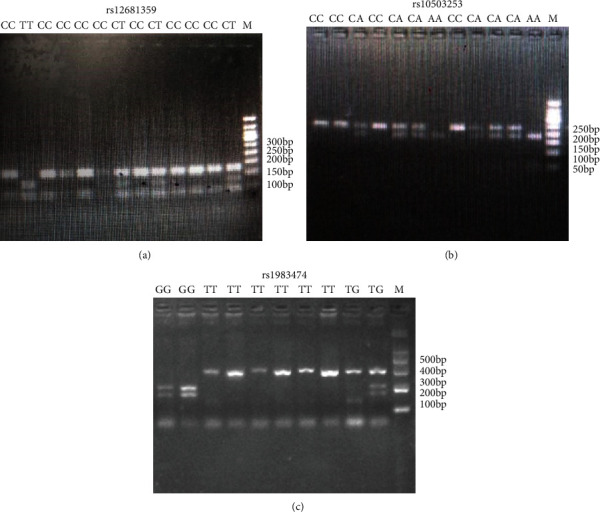
Polymorphism for rs12681349 (CC/CT/TT), rs10503253 (CC/CA/AA), and rs1983474 (GG/TG/TT) was genotyped by PCR-RFLP.

Polymorphism for rs12681349, rs10503253, and rs1983474 was genotyped by PCR-RFLP (Figure 1). PCR primers were designed using Primer5.0 and synthesized by Sangon Biotech (Shanghai) Co., Ltd.  Table 2 details the PCR primer sequence, PCR reaction conditions, and enzyme digestion conditions. Each 25 ul PCR reaction mixture included 0.5 ul upstream PCR primer and 0.5 ul downstream primer, 3.0 ul DNA template, 12.5 ul for PCR Master Mix, and 8.5 ul double-steamed water. The initial temperature of the PCR procedure was 95°C for 3 minutes, and the final step lasted for 5 minutes at 72°C. PCR products were observed by 2% agarose gel electrophoresis (AGE) under ultraviolet light. Enzyme digestion of the PCR products was done in a total volume of 15 ul as follows: 10 ul PCR products, 0.5 ul restriction enzyme, 2.0 ul Buffer solutions, and 2.5 ul double-steamed water. Samples were incubated in a 37°C water bath for at least 4 hours. The hydrolysis products were separated by 2.5% AGE. 15 samples of each genotype for each locus were sequenced to validate our results.

### 2.5. Statistical Analysis

Age and gender may be two basic characteristics that influence the disease phenotype. Considering the relationship between age of onset and gender of Parkinson's disease, subjects were stratified according to the average age of onset and gender to better analyze the relationship between *CSMD1* gene and the age of onset and gender of Parkinson's disease. Based on the age of onset, the included cases were divided into the early-onset PD group (EOPD, ≤50 years old), the late-onset PD group (LOPD, >50 years old), and corresponding age-matched control groups; based on the gender of onset, they were grouped into male PD or female PD groups, as well as the corresponding control groups. *T*-test and chi-square test were used, respectively, to evaluate the significance of age and gender differences between PD and control groups. The genotypes, allele, and genetic model frequencies of each polymorphism were calculated by chi-square test. After adjusting for possible covariates, including age and gender, odds ratio (OR) and 95% confidence interval (95% CI) were applied by logistic regression analysis to evaluate the relationship between the SNPs of *CSMD1* gene and PD risk. All data analysis was based on double-tail probability and calculated using SPSS 22.0 (BMI, Chicago, USA), and *p* values of less than 0.05 were considered statistically significant. ROC (receiver operating curve) was plotted using specificity versus sensitivity. The closer the curve follows the top left-hand border, the more accurate the test is. Area under curve and *p* values were computed using *z* statistics. The area under the curve is a measure of test accuracy.

The false-positive report probability (FPRP) was used to validate significant results [17, 18]. We set the FPRP threshold to 0.2. With a prior probability of 0.1, a significant result with an FPRP value less than 0.2 is considered a noteworthy finding. Moreover, statistical power was conducted by using PS Power and Sample Size software. Power ≥0.8 indicates that our sample size is still able to support our conclusion. On the contrary, if power <0.8, it indicates that the sample size needs to be increased.

## 3. Results

**Figure 2:**
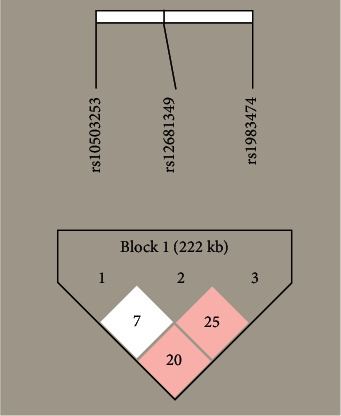
Analysis of linkage disequilibrium between case group and control group.

All the polymorphisms studied followed the Hardy-Weinberg equilibrium (Table 3) and no linkage disequilibrium (LD) (Figure 2) was observed in either PD patients or controls. Genotypes frequencies were significantly different between cases and control groups for rs10503253 (CA versus CC : OR = 1.544, 95% CI = 1.169–2.066, *p*=0.002) and rs1983474 (GG versus TT : OR = 0.599, 95% CI = 0.401–0.895, *p*=0.012) (Table 3). However, we failed to observe any association for rs12681349 between PD cases and healthy controls, which is consistent with a previous study on Iranians. In addition, no statistical differences were found in the haplotype allele analysis for *CSMD1* gene (Table 4).

Reviewing the polymorphism of rs10503253, genotype frequency analysis under different genetic models showed that there was a significant correlation in rs10503253 dominant model (AA + AC versus CC : OR = 0.677, 95% CI = 0.517–0.886, *p*=0.004) with the risk of PD. In the allele model, allele A reduced the risk of PD (A versus C : OR = 0.776, 95% CI = 0.623–0.996, *p*=0.046). In addition, the frequency of A allele (21.3%) was lower in PD than in the corresponding control groups (26.1%) within LOPD (OR = 0.766, 95% CI = 0.606–0.969, *p*=0.026). In the female cohort, allele A may be a protective factor of PD (OR = 0.687, 95% CI = 0.497–0.952, *p*=0.023) (Table 5).

For the rs1983474, there was a statistical significance in the analysis of the recessive model (GG versus TG + TT : OR = 1.837, 95% CI = 1.287–2.620, *p*=0.001). The results of genotype frequency analysis for different subgroups showed that the correlation of rs1983474 was significant for LOPD (TG versus TT: *p*=0.047; GG versus TT: *p*=0.042), male (TG versus TT *p*=0.015), and female (GG versus TT *p*=0.003) (Table 6). Further analysis suggested that the G allele gene frequency (48.7%) was higher than that of the control group (39.3%) in female queue, and G allele gene might be a risk factor for PD sensitivity (OR = 1.471, 95% CI = 1.118–1.935, *p*=0.006). We failed to find any correlations under all of the genetic models for rs12681349 (Tables 3 and 7).

**Figure 3:**
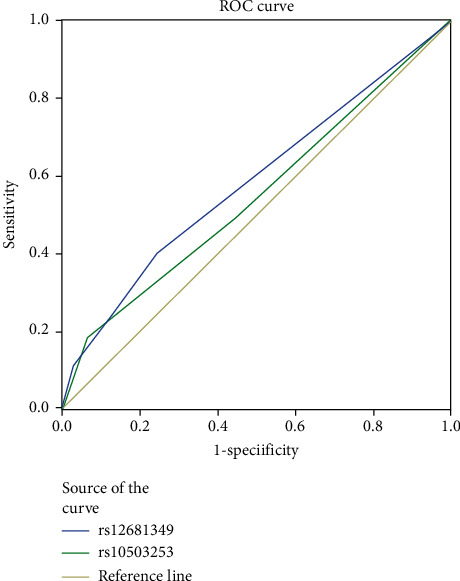
ROC for rs12681349 and rs10503253 in the diagnosis of Parkinson's disease were compared. ROC: receiver operating curve, *p* value based on z statistics. *p* > 0.05 was considered as significant. Curve toward upper left corner indicates better performance.

For the assessment of the use of CSMD1 gene as a diagnostic test for PD, receiver operating curve (ROC) analysis was performed (Figure 3). Area under curve, *p* value, specificity, and sensitivity were computed for variables including rs1983474 and rs10503253, except for rs12681349. The area *A*_*z*_ under the curve of rs1983474 and rs10503253 is 0.585 and 0.548, and the *p* value is *P* < 0.001 and *P*=0.013, respectively. ROC showed that rs1983474 and rs10503253 have a low diagnostic ability in Parkinson's disease.

Results of FPRP and statistical power analysis for significant findings are shown in Table 8. With the prior probability of 0.1, the meaning results of rs10503253 (CA versus CC (FPRP: 0.051), LOPD (FPRP: 0.077), and CC versus AA + CA (FPRP: 0.069)) are still noteworthy. Moreover, the results of the recessive model (TT + TG versus GG : FPRP = 0.051) and female (T versus C : FPRP = 0.086) of rs1983474 are also significant. The power for CA versus CC (genotype frequency, female cohort, and LOPD cohort) of rs10503253 is ＞0.8 (0.995, 0.859, and 0.915), and the power for TT + TG versus GG and male cohort (TG versus TT) of rs1983474 is also ＞0.8 (0.822 and 0.985), which showed that our data was capable of supporting our conclusion.

## 4. Discussion

The progression of Parkinson's disease seriously affects the patients' mobility and reduces the quality of life. Nevertheless, some negative risk factors, such as smoking, caffeine, and elevated serum urate levels [19–21], were instead thought to be protective, which were quite incredible for patients. Parkinson's disease is a complex, multifactorial disease, but the exact etiology is still unknown. In the last decades, many genetic variants tied up with Parkinson's disease have been identified, such as alpha-synuclein (*SNCA*), leucine-rich repeat kinase 2 (*LRRK2*), p10-induced putative kinase 1 (*PINK1*), DJ-1, parkin (*PARK2*), and ATPase type 13A2 (*ATP13A2*), leading to a better understanding of the complexity of its genetic patterns. Meanwhile, several pathogenic pathways have been identified, including accumulation of abnormal or misfolded proteins, increased oxidative stress, mitochondrial dysfunction [22], impaired ubiquitin-proteasome function, autophagic lysosome and mitotic phagocytic failure, loss of synaptic exocytosis and endocytosis [10], and endosomal transport [23].

GWAS is only the first step in identifying disease genes. Specific causal variations and their interactions at risk sites in related genes must be identified to fully understand their impact on PD development. Saeed [11] has recently identified that *CSMD1* is a significantly associated gene for PD in a GWAS. However, the relationship between *CSMD1* SNPs and PD has not been explored so far. Three selected SNPs of *CSMD1* gene were the protagonists of this study, including rs10503253, rs12681349, and rs1983474. The rs10503253 SNP was reported as an important genome-wide mutation in schizophrenia by Schizophrenia Psychiatric Genome-Wide Association Study (GWAS) Consortium [24]. The risk “A” allele of rs10502353 was associated with a neurocognitive function such as poorer performance on measures of general cognitive ability, visuospatial memory, and strategy formation, but not with emotional decision-making [13, 24]. Our results show that *CSMD1* gene variant rs10503253 (CA versus CC: *p*=0.002) was significantly associated with Parkinson's disease, but allele “A” reduced the risk of PD (OR = 0.776, 95% CI = 0.623–0.996, *p*=0.023). Furthermore, the population in northern China showed visible bias in the dominant model of the late-onset cohort of rs0503253. Allele “A” may be a protective factor of PD in the female cohort of rs10503253. However, more repeated linkage studies are required to conﬁrm this result. Another variant in *CSMD1*, rs12681349, was identified as a novel Parkinson's disease locus by stratified GWAS [25], which was analyzed in relation to Parkinson's disease in Iranian population [14], but no positive results were found. This finding supports our research result. The SNP rs1983474 of *CSMD1* has been revealed to be connected with variation in urinary C-telopeptide of type II collagen (u CTX-II) levels [15] in osteoarthritis which was a significant independent predictor of falls in patients with PD [26]. In this paper, we found significant differences in genotype frequency (GG versus TT: *p*=0.012), recessive model (GG versus TT + TG: *p*=0.001), LOPD (TG versus TT: *p*=0.047; GG versus TT: *p*=0.042), male (TG versus TT *p*=0.015), and female cohort (GG versus TT *p*=0.003) for rs1983474. This seems to support the role of inflammation in the pathophysiology of Parkinson's disease. However, the power analysis for allele frequency (power <0.8) of the above SNPs suggested that an investigation using a larger sample size is warranted to further analyze the associations.

The *CSMD1* gene is robustly expressed mainly in the developing central nervous system (CNS) and epithelial tissues [8], with some expression in the testicles, lung, breast, colon, thyroid, and pancreas [27]. *CSMD1* is an inhibitor of classical but not alternative complement activation pathways and a regulator of CNS inflammation, which is involved in the function of growth cone. Complement cascades play a crucial role in defending against pathogens in the immune system and also are relevant to synaptic plasticity and synaptic pruning [28], which seemed to be an especially important part for cognitive processes in the development of brain [29]. Steen et al. [30] demonstrated that genetic ablation of *CSMD1* in mice leads to behaviors associated with sluggish emotional responses, anxiety, and depression; results also indicated that *CSMD1* influenced the psychopathology of negative symptom spectrum. At the same time, genomic studies have indicated that *CSMD1* is implicated in several neurological related diseases like schizophrenia [24], cognitive impairment [9], multiple sclerosis [31], Alzheimer's Disease [32, 33], and familial Parkinson's disease [34], which provided strong support for the hypothesis that the *CSMD1* KO mice will exhibit neuropsychological deficits [30]. According to the previous studies, *CSMD1* gene has recently been linked to a variety of pathological processes ranging from cancer and psychiatric disorders to neurodegenerative diseases, and they are related to each other. In a Danish study, melanoma patients had a 44 percent increased risk of developing PD [35].

In recent years, pathogenic mutations associated with familial and sporadic PD account for only a small percentage of PD in most populations. Therefore, it is of scientific significance to explore risk factors of genetic susceptibility to PD. Noncanonical complement inhibitors related to complement action and/or independent function have emerged under many physiological and pathological conditions, which have potential application value as new disease biomarkers for neurodegenerative diseases. In our study, we came to the conclusion that rs10503253 and rs1983474 polymorphism of *CSMD1* may lead to PD. Those results support our previous hypothesis that *CSMD1* may be a candidate gene for idiopathic Parkinson's disease. This finding provides a reference for further researches on PD susceptibility genes. Certainly, there are also some limitations in our study. For example, all the samples were from the same medical center, and the selection bias resulted from the selection limitation. The study of the relationship between Parkinson's disease and Han population in northern China does not explain the effect of ethnic differences. Multicenter, large sample, and multiethnic studies may better explore the mechanism of *CSMD1* gene on Parkinson's disease. Further analysis of the mechanism of *CSMD1* gene in PD patients will be necessary to better understand the etiology and pathogenesis of PD.

## 5. Conclusion

This study is the first case-control study on the association of SNPs in the *CSMD1* gene and PD susceptibility in Han population of northern China. Our results show that polymorphisms in the *CSMD1* gene are closely related to PD. There were significant differences in rs10503253 and rs1983474 polymorphisms between PD cases and controls. However, the small sample size is the limitation of this study. Studies in larger populations and other ethnic groups are needed to confirm the correlation between *CSMD1* polymorphism and PD.

## Figures and Tables

**Table 1:** Demographic characteristics of PD group and control group.

Variable	PD (*n* = 423)	Control (*n* = 465)	*p*
	No. (%)	No. (%)	
Age (mean ± SD)			
All	64.1 ± 9.2	63.2 ± 9.7	0.216
Male	64.3 ± 9.9	63.4 ± 9.9	0.350
Female	63.9 ± 8.4	63.1 ± 9.5	0.421

Sex			
Male	224 (53.0)	246 (52.9)	—
Female	199 (47.0)	219 (47.1)	0.988

Age at onset of PD			
≤50	42 (9.9)	66 (14.2)	—
＞50	381 (90.1)	399 (85.8)	0.052

PD: Parkinson's disease; SD: standard deviation.

**Table 2:** Specific information on the PCR-RFLP reaction process of the three SNPs of CSMD1.

Polymorphisms	Primer sequences (5 ⟶ 3)	PCR conditions (°C/s)			Restriction enzyme digestion	Allele	DNA fragment size (bp)
		Denature	Extension	Annealing			
rs12681349	F-TTTGGATGGCAATTATCTACTGC	94/30	60/30	72/60	MseI	C	169
	R-ATAGATAATGCATGCTGGGCT					T	121 + 48

rs10503253	F-AGCAGGTTCAACAGACTTATTTC	94/30	60/30	72/60	MseI	C	221
	R-TCAGAGAAAGCCCTAGTCCC					A	171 + 50

rs1983474	F-GCTATCTGGGCAACCCTTAC	94/30	65/30	72/60	HincII	T	327
	R-TTTCAGCTTCCCTCCACTTAC					G	34 + 293

**Table 3:** Distributions of genotypes and alleles at rs12681349, rs10503253, and rs1983474 among Chinese with PD patients and healthy controls.

SNP		PD (%)	Control (%)	*p*	OR (95% CI)
rs12681349 (C＞T)					
Genotype frequency	CC	309 (73.0)	351 (75.5)	—	—
	CT	99 (23.4)	101 (21.7)	0.537	0.905 (0.659–1.243)
	TT	15 (3.5)	13 (2.8)	0.493	0.767 (0.359–1.638)

Allele frequency	C	717 (84.8)	803 (86.3)	—	—
	T	129 (15.2)	127 (13.7)	0.340	0.879 (0.674–1.146)

Dominant model	TT + CT	114 (27.0)	114 (24.5)	—	—
	CC	309 (73.0)	351 (75.5)	0.435	1.128 (0.834–1.525)

Recessive model	TT	15 (3.5)	13 (2.8)	—	—
	CC + CT	408 (96.5)	452 (97.2)	0.530	1.274 (0.598–2.713)

HWE	—	*p*=0.052	*p*=0.089	—	—

rs10503253 (C＞A)					
Genotype frequency	CC	268 (63.4)	251 (54.0)	—	—
	CA	127 (30.0)	184 (39.6)	0.002	1.554 (1.169–2.066)
	AA	28 (6.6)	30 (6.5)	0.649	1.135 (0.658–1.957)

Allele frequency	A	183 (21.6)	244 (26.2)	—	—
	C	663 (78.4)	686 (73.8)	0.046	0.776 (0.623–0.996)

Dominant model	AA + CA	155 (36.6)	214 (46.0)	—	—
	CC	268 (63.4)	251 (54.0)	0.004	0.677 (0.517–0.886)

Recessive model	AA	28 (6.6)	30 (6.5)	—	—
	CC + CA	395 (93.4)	435 (93.5)	0.897	1.036 (0.607–1.768)

HWE	—	*p*=0.019	*p*=0.630	—	—

rs1983474 (T＞G)					
Genotype frequency	TT	134 (31.7)	152 (32.7)	—	—
	TG	197 (46.6)	245 (52.7)	0.311	1.170 (0.864–1.584)
	GG	92 (21.7)	68 (14.6)	**0.012**	0.599 (0.401–0.895)

Allele frequency	T	465 (55.0)	549 (59.0)	—	—
	G	381 (45.0)	381 (41.0)	0.084	0.847 (0.702–1.022)

Dominant model	GG + TG	289 (68.3)	313 (67.3)	—	—
	TT	134 (31.7)	152 (32.7)	0.914	1.016 (0.763–1.353)

Recessive model	GG	92 (21.7)	68 (14.6)	—	—
	TT + TG	331 (78.3)	397 (85.4)	**0.001**	1.837 (1.287–2.620)

HWE	—	*p*=0.223	*p*=0.054	—	—

SNP: single nucleotide polymorphisms; HWE: Hardy–Weinberg equilibrium; OR: odds ratio; 95% CI: 95% confidence interval; CI: confidence interval; *p* > 0.05 was statistically significant, which is given in bold.

**Table 4:** Haplotype allele analysis of CSMD1 gene.

Haplotypes	Cases ratios	Controls ratios	Chi-square	*p* value
CCT	0.371	0.378	0.099	0.7527
CCG	0.302	0.274	1.739	0.1873
ACT	0.131	0.150	1.284	0.2572
ACG	0.054	0.063	0.654	0.4188
CTG	0.053	0.060	0.409	0.5227
CTT	0.041	0.040	0.005	0.9447
ATG	0.029	0.016	3.756	0.0526
ATT	0.018	0.019	0.007	0.9323

*p* > 0.05 had no statistical significance.

**Table 5:** Distributions of rs10503253 polymorphism in different subcomponent types.

rs10503253	Genotype	PD (%)	Control (%)	*p*	OR (95% CI)
Male					
	CC	139 (62.1)	137 (55.7)	—	—
	CA	66 (29.5)	90 (36.6)	0.090	1.480 (0.941–2.326)
	AA	19 (8.5)	19 (7.7)	0.169	0.615 (0.307–1.230)
	C	344 (76.8)	364 (74.0)	—	—
	A	104 (23.2)	128 (26.0)	0.320	1.163 (0.864–1.567)

Female					
	CC	129 (64.8)	114 (52.1)	—	—
	CA	61 (30.7)	94 (42.9)	**0.008**	1.740 (1.156–2.620)
	AA	9 (4.5)	11 (5.0)	0.472	1.400 (0.560–3.505)
	A	79 (19.8)	116 (26.5)	—	—
	C	319 (80.2)	322 (73.5)	**0.023**	0.687 (0.497–0.952)

EOPD					
	CC	25 (59.5)	36 (53.7)	—	—
	CA	13 (31.0)	26 (38.8)	0.507	1.335 (0.568–3.139)
	AA	4 (9.5)	5 (7.5)	0.930	0.935 (0.213–4.116)
	C	63 (75.0)	98 (73.1)	—	—
	A	21 (25.0)	36 (26.9)	0.760	1.102 (0.590–2.058)

LOPD					
	CC	242 (63.8)	215 (54.0)	—	—
	CA	114 (29.9)	158 (39.7)	**0.004**	1.570 (1.159–2.126)
	AA	24 (6.3)	25 (6.3)	0.594	1.174 (0.651–2.117)
	A	162 (21.3)	208 (26.1)	—	—
	C	598 (78.7)	588 (73.9)	**0.026**	0.766 (0.606–0.969)

EOPD: early-onset Parkinson's disease; LOPD: late-onset Parkinson's disease; OR: odds ratio; CI: confidence interval; *p* > 0.05 was statistically significant, which is given in bold.

**Table 6:** Distribution of rs1983474 polymorphism in different subcomponent types.

rs1983474	Genotype	PD (%)	Control (%)	*p*	OR (95% CI)
Male					
	TT	77 (34.4)	77 (31.3)	—	—
	TG	107 (47.8)	132 (53.7)	**0.015**	2.160 (1.162–4.016)
	GG	40 (17.9)	37 (15.0)	0.674	1.126 (0.649–1.954)
	T	261 (58.3)	286 (58.1)	—	—
	G	187 (41.7)	206 (41.9)	0.968	1.005 (0.776–1.303)

Female					
	TT	57 (28.6)	75 (34.2)	—	—
	TG	90 (45.2)	116 (53.0)	0.999	1.000 (0.640–1.563)
	GG	52 (26.1)	28 (12.8)	**0.003**	0.418 (0.234–0.746)
	G	194 (48.7)	172 (39.3)	—	—
	T	204 (51.3)	266 (60.7)	**0.006**	1.471 (1.118–1.935)

EOPD					
	TT	15 (35.7)	38 (56.7)	—	—
	TG	23 (54.8)	22 (32.8)	**0.021**	0.358 (0.150–0.855)
	GG	4 (9.5)	7 (10.4)	0.677	0.739 (0.177–3.074)
	T	53 (63.1)	98 (73.1)	—	—
	G	31 (36.9)	36 (26.9)	0.118	0.628 (0.350–1.127)

LOPD					
	TT	119 (31.2)	114 (28.6)	—	—
	TG	174 (45.7)	223 (56.0)	**0.047**	1.393 (1.004–1.934)
	GG	88 (23.1)	61 (15.3)	**0.042**	0.643 (0.420–0.985)
	T	412 (54.1)	451 (56.7)	—	—
	G	350 (45.9)	345 (43.3)	0.304	0.900 (0.737–1.100)

EOPD: early-onset Parkinson's disease; LOPD: late-onset Parkinson's disease; OR: odds ratio; CI: confidence interval. *p* > 0.05 was statistically significant, which is given in bold.

**Table 7:** Distributions of rs12681349 polymorphism different subcomponent types.

rs12681349	Genotype	PD (%)	Control (%)	*p*	OR (95% CI)
Male					
	CC	163 (72.8)	187 (76.0)	—	—
	CT	54 (24.1)	54 (22.0)	0.549	0.876 (0.569–1.350)
	TT	7 (3.1)	5 (2.0)	0.447	0.635 (0.198–2.043)
	C	380 (84.4)	428 (87.0)	—	—
	T	68 (15.2)	64 (13.0)	0.339	0.836 (0.578–1.208)

Female					
	CC	146 (67.6)	169 (77.2)	—	—
	CT	45 (20.8)	42 (19.2)	0.793	0.940 (0.589–1.498)
	TT	8 (11.6)	8 (3.7)	0.812	0.885 (0.324–2.419)
	C	337 (84.7)	380 (86.8)	—	—
	T	61 (15.3)	58 (13.2)	0.389	0.843 (0.572–1.243)

EOPD					
	CC	31 (73.8)	58 (86.6)	—	—
	CT	10 (23.8)	8 (11.9)	0.483	0.710 (0.272–1.849)
	TT	1 (2.4)	1 (1.5)	0.569	0.436 (0.025–7.585)
	C	72 (85.7)	124 (92.5)	—	—
	T	12 (14.3)	10 (7.5)	0.104	0.484 (0.199–1.176)
LOPD					
	CC	278 (73.0)	293 (73.6)	—	—
	CT	89 (23.4)	93 (23.4)	0.700	0.936 (0.669–1.310)
	TT	14 (3.7)	12 (3.0)	0.588	0.804 (0.365–1.769)
	C	645 (84.6)	679 (85.3)	—	—
	T	117 (15.4)	117 (14.7)	0.717	0.950 (0.719–1.254)

EOPD: early-onset Parkinson's disease; LOPD: late-onset Parkinson's disease; OR: odds ratio; CI: confidence interval.

**Table 8:** Results of false-positive report probability analysis for significant findings.

Genotype and variables	OR (95% CI)	*p* value	Statistical power^a^	Prior probability				
				0.25	0.1	0.01	0.001	0.0001
rs10503253 (C＞A)								
CA versus CC	1.554 (1.169–2.066)	0.002	0.995	0.018	0.051b	0.372	0.857	0.984
C versus A	0.776 (0.623–0.996)	0.046	0.390	0.136	0.321	0.839	0.981	0.998
CC versus AA + CA	0.677 (0.517–0.886)	0.004	0.795	0.024	0.069b	0.449	0.892	0.988

CA versus CC								
Female	1.740 (1.156–2.620)	0.008	0.859	0.091	0.232	0.768	0.971	0.997
LOPD	1.570 (1.159–2.126)	0.004	0.915	0.027	0.077b	0.477	0.902	0.989

C versus A								
Female	0.687 (0.497–0.952)	0.023	0.405	0.112	0.275	0.807	0.977	0.998
LOPD	0.766 (0.606–0.969)	0.026	0.380	0.082	0.212	0.748	0.968	0.997

rs1983474 (T＞G)								
GG versus TT	0.599 (0.401–0.895)	0.012	0.603	0.110	0.270	0.803	0.976	0.998
TT + TG versus GG	1.837 (1.287–2.620)	0.001	0.822	0.018	0.051	0.372	0.857	0.984

TG versus TT								
Male	2.160 (1.162–4.016)	0.015	0.985	0.265	0.519	0.922	0.992	0.999

rs1983474 (T＞G)								
GG versus TT	0.599 (0.401–0.895)	0.012	0.603	0.110	0.270	0.803	0.976	0.998
TT + TG versus GG	1.837 (1.287–2.620)	0.001	0.822	0.018	0.051	0.372	0.857	0.984

TG versus TT								
Male	2.160 (1.162–4.016)	0.015	0.985	0.265	0.519	0.922	0.992	0.999
EOPD	0.358 (0.150–0.855)	0.021	0.480	0.435	0.698	0.962	0.996	1.000
LOPD	1.393 (1.004–1.934)	0.047	0.658	**0.176**	0.390	0.876	0.986	0.999

GG versus TT								
Female	0.418 (0.234–0.746)	0.003	0.561	**0.142**	0.333	0.846	0.982	0.998
LOPD	0.643 (0.420–0.985)	0.042	0.459	0.227	0.468	0.906	0.990	0.999

T versus G								
Female	1.471 (1.118–1.935)	0.006	0.535	**0.030**	**0.086**	0.508	0.912	0.991

OR: odds ratio; CI: confidence interval. ^a^Statistical powers were calculated using the number of observations in the subgroup and the OR and *p* values. “b” denotes noteworthy findings are highlighted.

## Data Availability

All data generated or analyzed during this study are included within this article.
